# International Exploration of Health and Care Professionals Advanced Level of Practice in Intellectual Disability and/or Autism, Applied Through a UK, Health Education England Lens: Scoping Review

**DOI:** 10.1111/jocn.70494

**Published:** 2026-08-11

**Authors:** Ben Appleby, Martin Bollard, Julian Barratt, Sharon Kempson

**Affiliations:** ^1^ University of Wolverhampton Wolverhampton UK; ^2^ Head of the Centre for Advancing Practice Birmingham UK

## Abstract

**Aim:**

To explore, identify and describe the journey for health and care professionals (HCPs) working towards developing advanced level practice in intellectual disability and autism.

**Background:**

The review proposes there is an acknowledged gap in understanding the identity and positionality of advanced practice for people with autism and/or a learning disability.

**Design:**

Scoping review.

**Methods:**

PRISMA‐ScR guidelines were used to report the review. The published protocol was registered with the Health Science Network.

**Data Sources:**

Five academic databases were searched from 1994 to 2024 to capture the historical and contemporary trajectory of developments in advanced practice. Data extracted was in reference to the four pillars of Advanced Practice: Education, Leadership and Management, Clinical Practice and Research.

**Results:**

Forty‐four papers provided new insights into activities of advanced practice. Advanced practice was identified in ‘Assessing and Managing Care’; ‘Working in Partnership’; ‘Interprofessional Collaborative Working’; ‘Knowledge, Attitude and Competence’; and ‘Role Evaluation, Models and Tools’.

**Discussion:**

Explored the context and development of advanced practice, the positionality in intellectual disability and autism and thirdly the impact of advanced practice for this population group.

**Conclusion:**

Drawing on international literature, this review identified the types of advanced practice activities, the challenges for Health Care Professionals and the impact in their provision and management of care. Highlighting also the gaps and opportunities to develop advanced practice in education, leadership, management, clinical practice and research.

**Implications for the Profession and/or Patient Care:**

In the absence of international advanced practice role comparators in this review, more research and workforce development is required to standardise a professional framework for this group of people.

**Impact:**

Provides new and nuanced understanding internationally, in the ‘what’, ‘where’ and ‘what type’ of advanced practice. Has developed a conceptual picture of the international evidence‐base and reported different types of impact in ‘Clinical Practice’, ‘Leadership’, ‘Education’ and ‘Practice Development and Research’.

**Reporting Method:**

We adhered to EQUATOR guidelines and used the PRISMA‐ScR extension for scoping reviews to report the review.

**Trial Registration:**

Open Science Framework: DOI: https://doi.org/10.17605/OSF.IO/RT5DW

**Patient or Public Contribution:**

N/A: a literature review.

## Key Points

- This scoping review explored advanced levels of practice through a Health Education England lens, to identify the ‘What’, ‘Where’ and the ‘Impact’ of health care professional activities across a variety of clinical and community settings internationally.
- Available evidence identified gaps and opportunities to develop advanced practice in education, leadership, management, clinical practice and research.
- To develop advanced practice internationally, the evidence‐base supports a focus on ‘assessing and managing care’, ‘working in partnership’, ‘interprofessional collaboration’, ‘knowledge and attitude’ and ‘practice development’.
- Using the Health Education England framework helped to position advanced practice internationally, offering a way forward for health care professionals working in this area of practice.

## 1 Introduction

The recent growth in advanced clinical practice roles has refocused the debate on the core skills and capabilities required locally and regionally to practice at an advanced level. *Reshaping the Workforce* (Nuffield Trust 2016) by the Nuffield Trust identified the many benefits advanced clinical practice brings to patients, practitioners and employers, but also recognised the lack of clarity around the advanced practitioner role. In England, Health Education England (HEE) (2017, 10) defines advanced practice as ‘a level of practice characterised by a high degree of autonomy and complex decision making’. To help standardise advanced practice, HEE introduced a capabilities framework, the *Multi‐professional framework for advanced clinical practice* (HEE 2017). Alongside this framework, advanced practice is seen as a level of practice applicable across all healthcare professions, for exercising a level of autonomy across four domains, referred to as the four pillars of practice: clinical practice; leadership and management; education; and research. Building on from this, Skills for Health and Health Education England (2020) provided a capabilities framework for advanced clinical practice healthcare professionals working with People with intellectual disability and/or autism covering four domains: personalised and collaborative working; health and wellbeing; personalised care and support; and risk, legislation and safeguarding. The move towards framing advanced practice as a specific academic award with associated practice‐based learning for role preparation offers a way forward to standardising the scope and practice for advanced practitioners.

Internationally, many countries are in different stages of developing these advanced roles as a key part of their nursing workforce (International Council of Nurses 2020). In Australia, similar pillars of practice exist to guide the advancement of practice with the addition of *Optimising Health Care* (Gardner et al. 2017). In Canada, the role of Clinical Nurse Specialist (CNS) and Nurse Practitioner (NP) have been the primary role pathways to promote advanced practice, more specifically through Nurse Practitioner roles with its legally protected title status (Canadian Nurses Association 2019). The Canadian Nurses Association frames advanced practice roles through a core set of competencies, in *Direct comprehensive care, Optimising health systems, Education, Research, Leadership and Consultation and collaboration competencies*. In the United States of America (USA), Advanced Practice Registered Nurse roles (APRN) are exercised through different types of roles, CNS, NP, Nurses, Midwives and Nurse Anaesthetists. Less clarity associated with advanced practice roles exist in Asia. A national survey in Singapore, for example, showed that whilst the expansion of advanced roles was popular, there was uncertainty in how advanced practice nurses perceive their practice, competency and readiness for role expansion (Fong Yeong Woo et al. 2019).

Advanced practice innovations have been in existence in more less‐represented fields of practice, such as intellectual disability and autism as exemplified through nursing (Bollard and Jukes 1999; Jukes and Bollard 2003) and evidenced more recently in acute care liaison nursing roles (Brown et al. 2012). However, the specific development of and positionality of advanced practice in this complex speciality area is less well understood (Mafuba et al. 2023); in comparison to well highlighted areas of advanced practice, such as emergency care or general practice (King et al. 2017).

This review was part of a funded piece of work for National Health Services England—Workforce Training and Education, and as such positionality refers to the four pillars of advanced practice highlighted earlier (HEE 2017). The starting point for this review is to propose there is an acknowledged gap in understanding the identity and positionality of advanced practice for people with autism and/or a learning disability. Therefore, the aim of this scoping review was to articulate the journey for health and care professionals (HCPs) working in this area of practice, internationally, identifying their contribution and using the HEE framework to position the scope and nature of advanced practice and the impact on care delivery.

### 1.1 Background

People with intellectual disability and/or autism have a range of complex health and social care needs. People with intellectual disabilities are known to experience significant health inequities (Hosking et al. 2016), have high co‐morbidity rates (Cooper et al. 2018; Heslop et al. 2014), including obesity (Biswas et al. 2010), epilepsy (Heslop et al. 2014), respiratory disease (O'Leary et al. 2018) and cardiovascular disease (Glover et al. 2017). These features of co‐morbidity place an emphasis on the importance of health promotion, surveillance and an educational role when caring for people with learning disabilities. Enhanced roles in this field have also focused on defined areas of practice matched to need, such as forensic, mental health and epilepsy (Mason and Phipps 2010; Dalgarno and Riordan 2014; Auberry and Cullen 2016). Like other areas of practice, working at an advanced practice level with this group of people demands capabilities, skills and knowledge enacted across the health system, to be able to demonstrate positive outcomes (WHO 2023). Despite the complexity of need associated with this group of people, relatively little is known about advanced practice developments in the field of intellectual disability and/or autism with a lack of extensive published work in this area (Bollard et al. 2025). Furthermore, locating specific examples of advanced practice in an international context for intellectual disabilities and autism care is challenging. This is compounded by ‘intellectual disability’ itself being a contentious term, a term that has never featured in diagnostic criterion‐based assessments for clinicians (Lovell 2021).

Internationally, there is an established body of evidence demonstrating HCPs operating at advancing levels to improve effectiveness in care, patient safety and person‐centred care (Laurant et al. 2018). However, much of the evidence‐base supporting effectiveness is from health professionals in a specific role, such as ‘Advanced Nurse Practitioner’ (Woo et al. 2017). Further, randomised controlled trial evidence demonstrates positive clinical and patient satisfaction outcomes from advanced practitioners working in primary care, outpatients, or specialist centres (Htay and Whitehead 2021). However, very little is known about the contribution practitioners make in advanced roles when working with people with intellectual disability and/or autism, with even less known regarding their impact. Therefore, a systematic scope of the literature will help provide identity and positioning for the journey of ‘Advancing Practice’ for People with intellectual disability and/or autism, irrespective of named professional role.

## 2 The Review

Our scoping review begins from an evidence‐informed positional standpoint that mapping the components of advanced practice in intellectual disability and/or autism is desirable and necessary for today's healthcare landscape, both for practice in the UK and to foreground work internationally. This is particularly relevant for practitioners working with people with intellectual disability and/or autism in extended roles. Scoping the literature has the potential to develop evidence‐informed global practice comparisons, once domain areas of care, against the four pillars of advanced practice are identified (HEE 2017).

## 3 Aim

Our aim was to review published literature to systematically explore, identify and describe the journey towards developing advanced practice and the impact on care delivery in intellectual disability and autism. Our intention was to address the following scoping review question: ‘What is the positionality, identity and impact of advanced practice for care delivery in intellectual disability and autism?’. The specific objectives were to:

1. *Review the context and development of advanced practice in intellectual disability and autism (International Evidence‐Base)*
2. *Explore the impact of advanced practice on health professionals' delivery of care for intellectual disability and autism (International Evidence‐Base)*
3. *Identify the underpinning identity/positionality of advanced practice for intellectual disability and autism in relation to knowledge, skills, behaviour and practice experiences*.

The intention was to capture the evidence‐base of ‘advanced practice’ across multi‐professional practitioners working in the discipline of intellectual disability and autism. The review focus was on advanced practice for nursing and allied health professionals; medical professionals who already contribute to advanced care in the management of care in autism and learning disability were excluded.

## 4 Methods

### 4.1 Design

Methods of Scoping Review (Munn et al. 2022; Khalil et al. 2016; Arksey and O'Malley 2005) were used to address the research problem and map the journey and impact of advanced practice in intellectual disability and autism. Scoping review methodology is seen as advantageous in comparison to traditional systematic review approaches that address narrower, empirically focused questions (Evans 2023). To address aims and objectives, in our scoping review we identified key concepts/definitions, characteristics and knowledge gaps in advanced practice, and the impact of advancing practice initiatives in domains of Advancing Practice.

The HEE (2017) conceptualisation of ‘advanced practice’ was used to map out and understand advanced practice in the literature. Wherein, empowered activities of care in leadership, research, education and practice were recognised as advanced practice activity. Using these approaches helped to identify ‘advanced practice’ for an international evidence‐base and provided a reference point for where HCPs sit against current guidance on the standardised recognition of advanced practice education and training (HEE 2017). Applying these indicators also helped to address our research problem and demonstrated a good fit with scoping review methodology: designed to map a broad range of literature against a specific topic, field, concept, or issue (Peterson et al. 2016; Munn et al. 2022).

To enhance rigour of this scoping review the latest PRISMA reporting guidance was applied (Tricco et al. 2018). Some PRISMA‐ScR 2020 recommendations were not used, such as the methods that relate to handling quantitative data which are not relevant in this scoping review. This scoping review focuses on drawing out an overview and conceptual understanding of advanced practice not necessarily defined by solely quantitative approaches, for example when meta‐analysis is used in quantitative aggregation of outcomes in systematic reviews.

### 4.2 Search Methods

A comprehensive electronic search was conducted by the research team (BA and SK), guided also by information specialists. Dates were limited from 1994 to 2024 to capture the historical and more contemporary trajectory of developments in advanced practice. Translated papers into English were included to enhance international representation.

#### 4.2.1 Systematic Search of Academic Literature

Five databases were searched: CINAHL, MEDLINE, PsycINFO; Sociology Index and World of Science, as provided sufficient scope for professional practice in this field of practice and were fully accessible to the research team. Table 1 identifies key word search terms using MESH and Free Text terms. Boolean logic operators OR was used to combine search terms in each search string and AND used for specificity, no further filters or limits were used. Forward (i.e., citation searching) and backward (i.e., checking of reference lists) chaining techniques from identified papers were employed (Booth 2009).

**TABLE 1:** Key word search terms.

Database	Search terms
Ovid MEDLINE, CiNAHL, Scop Index, Psych Info, WoS	Nurses or Specialist Nurse or health care professionals AND Allied Health Professionals AND 2 Learning Disabilities or Intellectual Disabilities or Mental Retardation or Learning Difficulties or Special Needs AND Autism or Autism Spectrum Disorders AND 3 Advanced Practice or Enhanced Practice or Clinical Practice AND healthcare or social care or hospital or health services

#### 4.2.2 Structured Search of the Grey Literature

A cyber‐attack at the British Library prevented a full search of Unpublished (‘grey’) literature (British Library 2024). However, we also used Google Scholar to catch any papers not currently indexed in chosen databases using ‘Advancing Practice’ and ‘Professional Terms’.

#### 4.2.3 Bibliographic Management

Our searching and screening process was recorded using the bibliographic data management system RefWorks. This provided an audit trail of decision making at each stage of screening.

### 4.3 Inclusion Criteria and Eligibility

Inclusion criteria were produced from 1994 onwards, English language, empirical studies, qualitative, quantitative, mixed methods and secondary empirical papers with a focus on advanced practice innovations or evaluations in intellectual disabilities and/or autism. Only since this time had advanced practice developments been more formally conceptualised and the last 20 years would enable a previous and contemporary capture within this field of practice. Exclusion criteria were opinion papers, protocols, editorials and non‐English papers (see Table 2).

**TABLE 2:** Inclusion and exclusion criteria.

	Inclusion	Exclusion
Population	Health Care Professionals, Nurses (all fields), Specialist Practitioners, Allied Health Professionals: Occupational Therapists, Physiotherapists, Pharmacists, Psychologists, Dietitians, Arts Therapists, Speech and Language Therapists, Orthoptists.	Medical Doctors, Health Care Scientists, Students; Undergraduate Education.
Exposure	Children and Adults with Learning Disability and/or Autism, Intellectual Disabilities.	ADHD, Infants.
	Any Practice, Education, Leadership, Research Advancing Practice Innovations, Evaluations.	Papers about the condition, Development of educational programmes, products or tools (but not yet applied).
Outcome	Any reported Practice (Care Delivery), applied Leadership, Education and Research.	Outcomes not related to Population or Practice, Leadership, Education and Research; Finance.
Study	Primary/Secondary or Descriptive and Policy‐based literature	Opinion papers, editorials, protocols, Non‐translatable to English papers.

A PEOS (Population, Exposure, Outcome, Studies) framework was used to determine eligibility for inclusion (see Table 2) and helped to identify and then filter the diverse studies and exposures to advanced practice for intellectual disability and autism. The subsequent refined criteria helped to determine the number of papers included at full text. In this scoping review, the use of the PEOS framework helped to provide a clear visual representation of components for this exploratory type question (Evans 2023); and for this reason, it was a preferred choice over other options such as Population, Concept and Context (Kestenbaum 2019) and SPIDER (Cooke et al. 2012). Using PEOS helped to clearly identify ‘who’ we were interested in (Population), the types of advanced practice and with whom (Exposure) and reported care delivery, leadership, education and research (Outcomes). Using PEOS in this way ensured the research terms were on the same page to uphold a vigorous screening process.

### 4.4 Search Outcome

A summary of the search process and reasons for exclusion is provided in a PRISMA‐Scp flow diagram (Tricco et al. 2018), Figure 1 and Table 3. As a quick overview, many papers reported descriptions of living with and being a recipient of care for intellectual disability and/or autism, but without reference to HCPs; other papers described the condition and management strategies without reference to HCPs.

**FIGURE 1:**
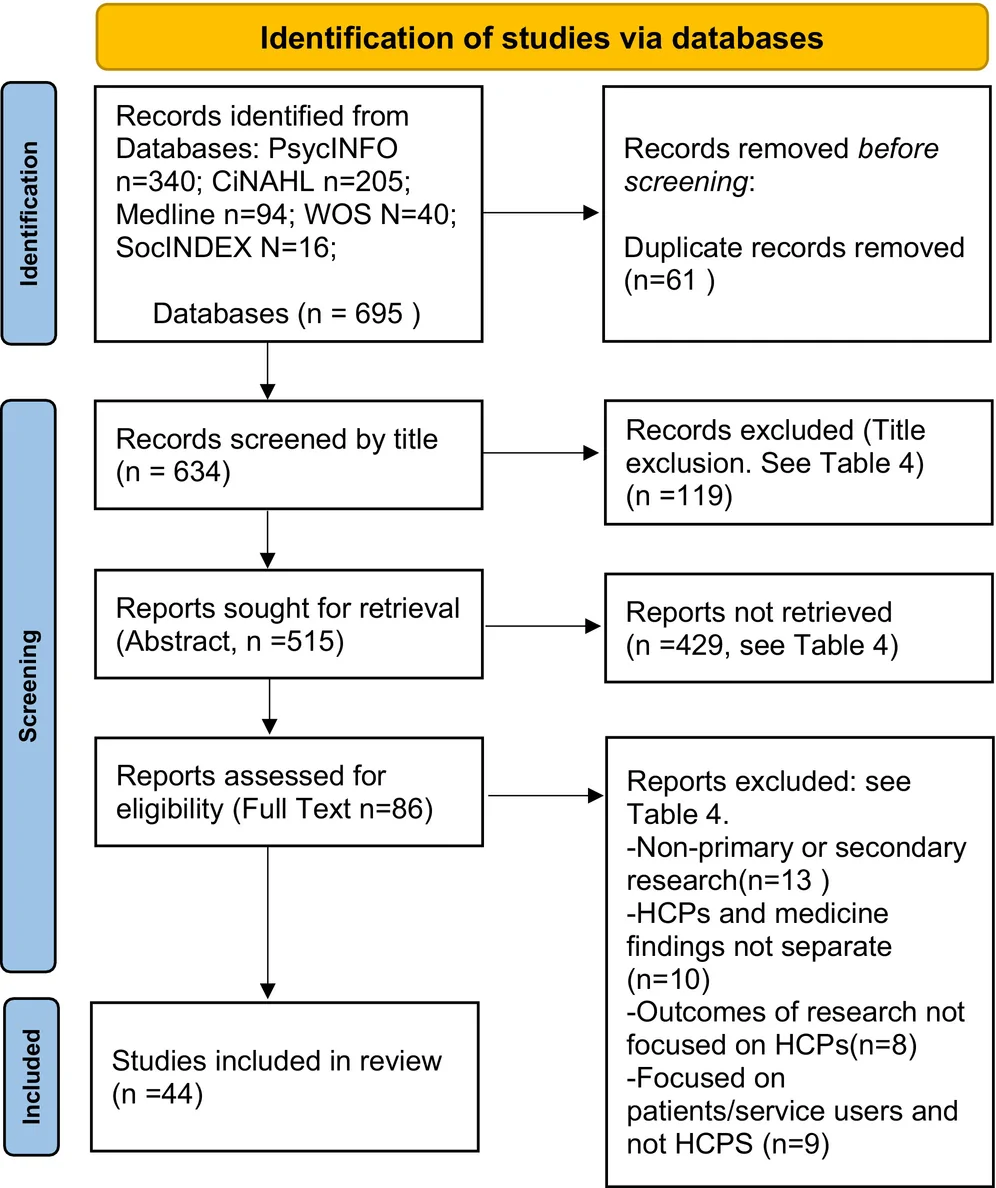
PRISMA flow diagram. [Colour figure can be viewed at wileyonlinelibrary.com]

**TABLE 3:** PRISMA application of exclusion criteria.

Stage of assessment	Reasons for exclusion
Excluded by title (not an obvious fit as per inclusion table)	‐Focused on MMR; practice development but not applied to practice; screening for conditions; how patient's function; impact of COVID; Medicine focused; focused on sexual behaviour; oral health knowledge, books not journal papers.
Excluded by Abstract (partly related to inclusion table, but missing information in title)	‐Evaluation usefulness of measurement scales; research with parents with autism; role of parents with ADHD; how HCP are viewed; practice development but not applied to practice; focused on ADHD not Autism or LD; focus on rural communities; patients access to services; patient focused; care‐giver focus not HCP; exploration of diagnosis but not HCPs; preparing students; psychometric properties of tools; testing measurement scales; focus on curricula education; infants not children; updating guidelines; validation of guidelines; not fitting with ‘advancing practice’; exploring staffing levels; pain assessment; HCPs but not working with LD or Autism; development of programmes but HCPs not directly involved; telehealth development but not involving HCPs; evaluating telehealth training package; pharmacology treatment; evaluation of service provision; RCT intervention but not involving HCPs; evaluation of technology but not applied to practice.
Excluded final full text check (see data extraction table—not clear in full text)	‐Non primary or secondary research; exploring pathways to reduce cost but not HCP focused; can't report separate ADHD/Autism outcomes; can't separate patients/parents views from HCPs; narrative review; planning for care not actual care; reports of attendance at educational programme.

### 4.5 Quality Appraisal

Due to the interpretive nature of the scoping review question and review aims, and based on the premise that scoping reviews aim to provide an overview or map of the evidence rather than an assessment of methodological limitations (Munn et al. 2018; Peters et al. 2015), it was not considered appropriate to exclude empirical studies on the basis of either design or study quality. For this reason, standard quality assessment was not undertaken for inclusion. Methodologically weak studies were considered equally relevant in addressing our scoping review question, with quality of included papers observed and commented in the reporting of the results and the discussion, using CASP tools as a reference point for observed comments on methodological quality. Observing study quality in this way helped to provide some perspective on the general level of empirical evidence reviewed, but strict criteria were not applied. In the results section, at the beginning of each theme and in the discussion in each theme, the quality of studies was observed, providing a clear methodological reference point to the type of evidence underpinning each theme.

### 4.6 Data Extraction

A bespoke data extraction table was created with standard headings for author, date, title, country of origin, journal, type of publication, aims, design, stated limitations and results (Table S1: Data Extraction Table). Categories of ‘advanced practice’ were contextualised against ‘What’, ‘Who’ and ‘Where’ advanced practice activity was taking place, using the Multi‐professional framework for advanced clinical practice in England (HEE 2017) as a guide to provide context for the type of advanced practice being developed, specifically in the areas of ‘clinical practice’, ‘leadership and management’, ‘education’ and ‘research’. The impact of advanced practice was also recorded against activities of clinical practice, leadership and management, education and research as the established four pillars of advanced practice (HEE 2017). These methods helped to order generated themes against the pillars of advanced practice, providing insight and context to actual activities that have been developing over a 30‐year period.

### 4.7 Synthesis

Meta‐summary (Dixon‐Woods et al. 2004; Finfgeld‐Connett 2018; Aveyard 2018) was used as a guide and method of synthesis. The process involved grouping similar studies to make collective sense of the complex data from different types of included literature (Aveyard 2018). Similar to a process of theme development in classic qualitative content analysis (Miles and Huberman 2013; Graneheim and Lundman 2003), the process involved (a) data extraction and interpreting the main focus of each paper; (b) exploring the relationship in and between studies, which involved grouping similar studies; and (c) assessing the robustness of the synthesis by reflecting on the value of synthesis methods in addressing the main aims of the study—producing insight into ‘what’, ‘where’ and ‘impact’ of advanced practice observed in grouped studies.

From the final inclusion of papers (*N* = 44), synthesis of outcomes across all included papers was examined collectively and organised to provide context to the type of advanced practice taking place, by whom and where (Figure 2). Activities were framed into ‘themes’ which represented pillars of ‘advanced practice’ using inductive interpretation of data to align and group HCP activities against named themes (Christmals and Gross 2017). Using this approach also helped to frame the journey of advanced practice activities against current understanding of the nature of advanced practice (HEE 2017) so also provides a reference point to the direction travelled from the early 1990s to November 2023.

**FIGURE 2:**
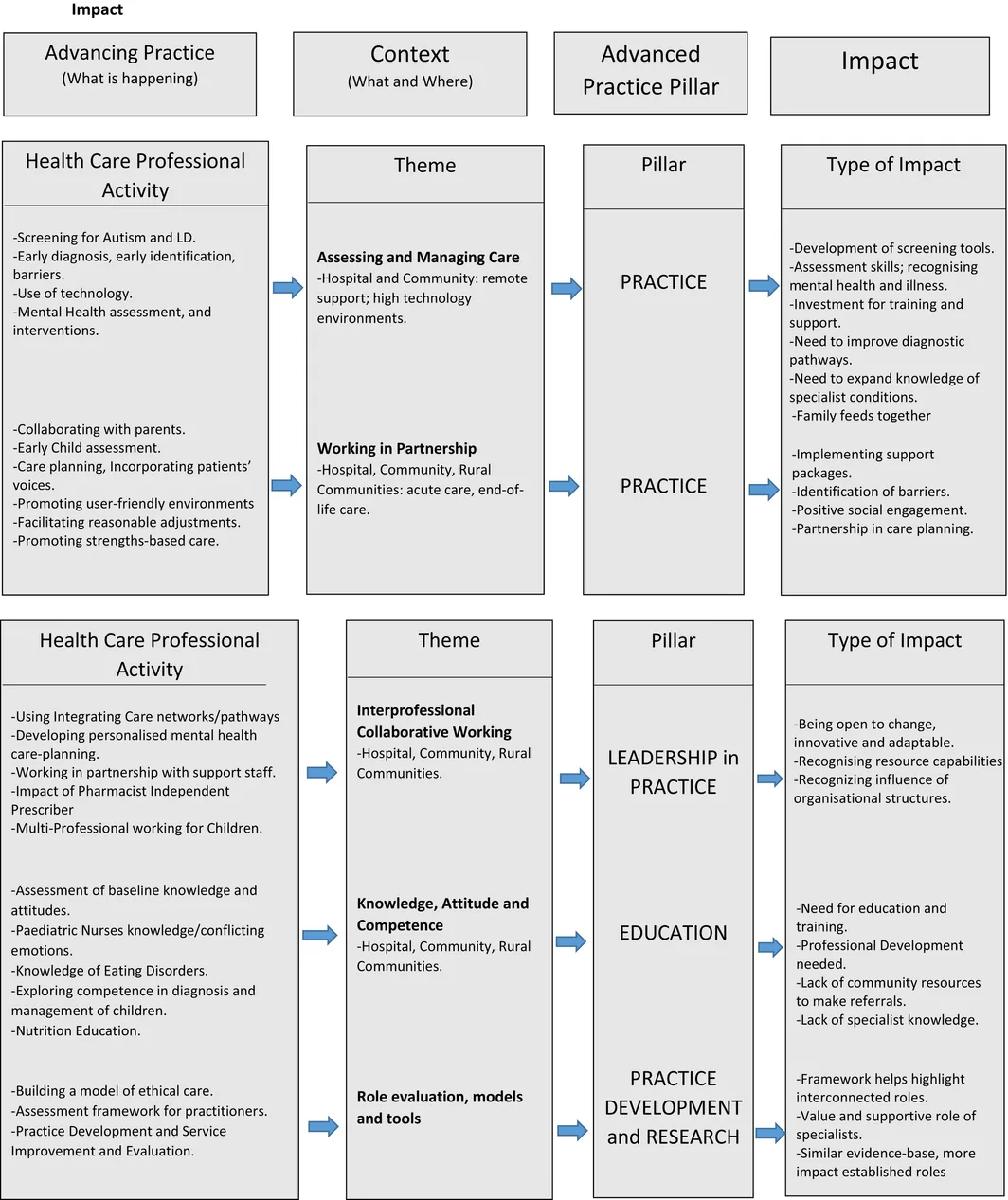
Synthesis of advancing practice: ‘What’, ‘Where’ and ‘Impact’. [Colour figure can be viewed at wileyonlinelibrary.com]

## 5 Results

The process of data synthesis produced several themes of ‘advanced practice’ (Figure 2). These categories helped to illustrate and explain the type of activity HCPs have been engaged in and the impact on their practice and care. Developing categories in this way helped to frame ‘What’, ‘Where’ and the ‘Impact’ of HCPs activities across a variety of clinical and community settings. As planned, categories were developed with reference to the pillars of advanced practice (HEE 2019), which helped to align actual activity with themes of capabilities noted in the Advanced Clinical Practice Framework. Results are reported against the themes developed. As would be expected when mapping and categorising studies in a scoping Review (Munn et al. 2018), not all papers fit neatly into each category. The main features, scope and outcomes of each paper are discussed in each category.

### 5.1 Characteristics of Included Papers

The included full text papers (*n* = 44) were international. All primary empirical studies were observational, using a variety of qualitative, quantitative and mixed methods. From the UK (*n* = 15), Ireland (*n* = 7), Netherlands (*n* = 4), Australia (*n* = 3), USA (*n* = 3), Canada (*n* = 2) and additional papers from India, Philippines, Brazil, Saudi Arabia, Switzerland, Iran, Sweden and New Zealand. There were (*n* = 10) secondary empirical studies using a variety of systematic methods, including scoping and integrative review methodologies. The included full text papers ranged from 1996 to 2024.

A key feature of the included papers and international evidence‐base is the reference to ‘Health Care Professionals’ (HCPs) and the activities they are engaged in, which relate to exploring and developing advanced practice. Across the international literature, HCPs engaged in ‘advanced practice’ activities are referred to as ‘HCPs’ and are not necessarily practising in a specialist role or with a designated advanced practitioner title. Therefore, in terms of capturing the journey of developing advanced practice and to address key aims of this review, all practice activities involving HCPs that explored concepts relative to the pillars of advanced practice—clinical practice, leadership and management, education and research (HEE 2017) were included.

To get a sense of the journey of how advanced practice has been developing, where logical, studies in each theme have been reported chronologically. The results are presented in the themes synthesised (Figure 2).

### 5.2 PRACTICE—Assessing and Managing Care

The type of evidence in the Practice Pillar and Assessing and Managing Care theme is varied. All papers are peer‐reviewed and largely use, report and execute established methods well. Primary empirical studies include, from New Zealand, a small‐scale ethnographic study (Taua and Farrow 2009), innovative survey design using vignettes from the Netherlands (Burke et al. 2016), in‐depth qualitative analysis in the UK (Daniels et al. 2021), telephone interviews in Australia (Johnsson et al. 2019), a case study approach in Saudi Arabia (Al Sharif and Ratnapalan 2016) and in Sweden a qualitative retrospective critical incident analysis (Pettersson et al. 2023). Secondary reviews included a systematic review from a Nurse Practitioner Australian perspective (Pridding and Procter 2008), a Canada‐based scoping review (Morris et al. 2019) and a scoping review from Ireland (Doody et al. 2019), all these reviews used established review methods. Empirical exploration using primary methods of research focuses on practice‐specific challenges in each country and can lack generalisation to other settings and countries. Likewise, the inclusion criteria for secondary reviews are limited by country in some reviews (Pridding and Procter 2008), but all other reviews offer international inclusion criteria and full reporting of methods.

Assessment and the management of care were important features of practice and took place in varied settings. The review by Pridding and Procter (2008) focused on Australian mental health nurses, highlighted the challenges in caring for and assessing people with an intellectual disability and personality disorder. The role of the Nurse Practitioner (NP) highlighted the need for enhanced communication skills and development of therapeutic relationships as core activities the Nurse Practitioner can use to enhance care delivery. Similarly, in their ethnographic observational study in New Zealand, Taua and Farrow (2009) highlighted the challenges of caring for patients with an intellectual disability and mental health problem. For advanced practice, Taua and Farrow (2009) emphasises the limited evidence‐base in nursing to guide good practice, suggesting ‘narrations’ of practice experience could help bridge the theory‐to‐practice gap. In this study, the lack of nursing models, overuse of ‘borrowed’ theories from mental health and lack of nursing‐focused assessment tools all were limitations. These examples highlight the need for empirical research in areas of complex assessment, in this example for advancing practice in mental health when HCPs are caring for intellectual disability patients with a dual diagnosis.

Burke et al. (2016), in the Netherlands, explored the ability of HCPs to accurately assess mental health problems in children as a function of their disability diagnosis. To assess diagnostic skills, HCPs (*n* = 431) completed an online survey, evaluating a series of vignettes describing children with symptoms of mental‐health problems. Vignettes varied by problem type Attention‐Deficit/Hyperactivity Disorder (ADHD), Generalised Anxiety Disorder (GAD), ASD, Conduct Disorder (CD) and Major Depressive Disorder. Results showed that recognition of mental‐health problems varies by condition type, with ADHD best recognised and GAD worst. For all conditions HCPs found it more difficult to diagnose in children rather than adolescents. The number of symptoms were also positively correlated with accurate assessment of mental health—particularly for recognition of ASD, reinforcing the need for education to improve practice in this area. Similarly, Daniels et al. (2021) qualitative study in the UK conducted 25 interviews to capture HCPs views and role related to the impact of the diagnosis process and the impact of this assessment on families. People with intellectual disability and/or autism have a high number of comorbidities, requiring multidisciplinary care and are at high risk of morbidity and preventable mortality, therefore provision of compassionate, person‐centred care is essential to prevent complications and avoid death (Brown et al. 2012).

The assessment process determining whether an individual has intellectual disability and/or autism, involves navigating different professionals across different healthcare settings. In the UK, many HCPs reported a struggle to navigate differing expectations of the diagnostic process between parents and clinicians, as well as managing objectivity in the face of significant distress (Daniels et al. 2021). This study highlighted the need for advanced collaborative skills and leadership in this area of practice when assessing and managing care, which the authors suggest points to a nuanced clinical practice feature of intellectual disability and/or autism advanced practice.

Several studies explored different ways of working in complex areas or cases wherein managing complexity is a feature of advanced practice. Johnsson et al. (2019) explored the feasibility, essential requirements and potential barriers for occupational therapists in delivering therapy support for ASD patients via video‐conferencing technology. Based in Australia, a multidisciplinary team of *n* = 16 allied health professionals were telephone‐interviewed. The effective use of technology was very much dependent on sufficient training and education, with success in delivering care through tele‐health dependent on the ‘role of collaboration’. The impact of the service was positive, with access to ‘autism specialist services’ highly valued. This study highlighted the therapeutic practice impact for Occupational Therapists, but it is unclear how other types of care (e.g., nursing, physiotherapy) would use this technology to enhance service delivery and care.

Morris et al. (2019) in their scoping review across 27 primary research studies captured HCPs experience of managing care. A key theme to emerge was ‘Complexity Beyond Usual Role’ which resulted in lack of confidence and clarity around roles of health care providers (Unigwe et al. 2017), and their ability to address complex needs (Holmes et al. 2014). In terms of advanced practice, these challenges are identified against those HCPs that are not necessarily working in an ‘advanced role’ such as practitioners with a speciality focus. Illustrating the challenges faced such as training needs and prior experience impacting on HCPs without significant experience working with patients with autism, across primary, secondary and tertiary care settings.

Similar training needs are identified by Gagnon et al. (2023), reporting the challenges encountered by various health professionals when intervening with individuals who present a comorbidity of ASD and gender dysphoria. Their systematic review illustrated that HCPs need to expand their knowledge on gender dysphoria to have a better understanding of the various challenges, such as discrimination. Reiterated earlier by Morris et al. (2019) demonstrated the need for HCPs to adapt their approach to the individual's unique profile to promote sensitive communication and collaboration. Communication and the development of communication strategies feature in the Multi‐professional capabilities framework (HEE 2017) and as a key domain in Skills for Health capabilities (Skills for Health and Health Education England 2020). Lovell and Skellern (2020) provided a good example of how sensitive communication and building effective therapeutic relationships can uncover how ‘personal history’ can relate to offending behaviour. This example featured forensic intellectual disability nurses, and for advanced practice illustrating that the core skill set of collaboration and building relationships are at the heart of best practice, despite the role of the HCP.

Al Sharif and Ratnapalan (2016) in Saudi Arabia explored how HCPs in Emergency Departments managed care for children with ASD, recommending collaboration with parents and consistency in health personnel and its central importance to help manage communication and care, as reflected in Domain C (Skills for Health and Health Education England 2020). However, promoting familiarity with consistent therapists organisationally could be difficult to achieve. Creating favourable conditions is also important in high‐technology environments to help HCPs collaborate with parents and promote effective work environments (Pettersson et al. 2023). The complexity of these environments demands advanced person‐centred collaborative skills, as highlighted in the multi‐professional capabilities framework (HEE 2017).

Focusing on Intellectual Disability Nurses, Doody et al.'s (2023) scoping review highlighted the limited evidence base underpinning such roles, specifically on assessment and intervention strategies. How Intellectual Disability Nurses provide care using the biopsychosocial‐educational model of care and the required values, behaviours and skills is discussed at length. But the actual impact of HCPs and their contribution to developing advanced practice from an empirical standpoint was limited.

For managing and assessing care, the evidence reviewed has demonstrated the varied types of HCPs delivering care and challenges in their role and to advancing practice. Internationally, in terms of workforce planning, there is clear recognition of the challenges faced in managing assessment. For HCPs in specific roles, their roles are better understood and have more confidence and competence in managing assessment and care. Ultimately, policy decisions for workforce development are based on evidence and as highlighted (Doody et al. 2023) an evidence‐base for impact is required to effectively develop advanced practice for optimising care delivery for patients and their carers.

### 5.3 PRACTICE—Working in Partnership

Evidence underpinning working in partnership is varied and has some crossover of findings relevant to the previous theme. Review papers used established methods of Integrative and Scoping Review methodology (Doody et al. 2019; Murthi et al. 2023; Noorlandt et al. 2020; Morris et al. 2019), and methods were described and defended. A potential limitation in these reviews is a lack of grey literature and strategies to find this type of literature. Use and access to databases is comprehensive. Primary studies were used to explore working in partnership in the UK by mixed methods (MacArthur et al. 2015), and a European‐based survey (Luzi et al. 2018); MacArthur et al. (2015) study provided good evidence of rigour, for example, through sampling and theme development (MacArthur et al. 2015), the survey by Luzi et al. (2018) identified a large target population, but response rate and questionnaire development was underreported.

Working in partnership across different areas of health and social care settings was integral features of practice. Activities included promoting both strength‐based care and user‐friendly environments with others.

MacArthur et al. (2015) in their UK mixed methods study examined the role of intellectual disability liaison nurses in helping to improve care and access to general hospital services for people with intellectual disabilities. Through partnership working, intellectual disability liaison nurses demonstrated the importance of facilitating reasonable and achievable adjustments to achieve person‐centred care, as a key feature of advancing their scope of intellectual disability practice (HEE 2017). Doody et al.'s (2019) integrative review explored experiences of adults with intellectual disability in the involvement of nursing care planning. From an advanced practice position, Doody et al. (2019) highlighted a clear need for intellectual disability nurses to highlight and demonstrate the involvement of adults with intellectual disability in care planning—as no studies in this review were identified to meet the inclusion criteria. This highlighted an obvious gap in empirical evidence and the need for HCPs involved in partnership working associated with planning care, to report their experiences. Also focused on partnership working, Murthi et al.'s (2023) scoping review proposed the use of a ‘strengths‐based’ approach to improve care. Of the 38 studies highlighted the importance of the patient's voices being integrated into occupational therapy practice in meaningful and purposeful ways. This study is a timely reminder that person‐centred assessment and building positive relationships should be a cornerstone of advancing practice and the necessary skills to do so sit beyond the standard scope of Intellectual Disability Nursing practice. A noted limitation of this review was the absence of culturally diverse literature; studies being limited by geography; studies also lacked representation of autistic communities from global majorities.

Noorlandt et al.'s (2020) scoping review explored Intellectual Disability (ID) patients in end‐of‐life care, and how they are involved in shared decision‐making about their care and treatment. Focusing on HCPs that ‘worked’ in community and residential care for adults, of the *n* = 10 papers that met the final inclusion only two papers described processes of decision‐making in which persons with ID actively participated, a practice feature of advanced personalised care and support (Skills for Health and Health Education England 2020). Furthermore, in only one paper, best practice and guidelines for decision‐making in palliative care for people with ID were defined. Clearly, with the very few papers (proportionally) that identify shared decision‐making, indicates a tangible need to improve practice in this challenging area of care provision. Morris et al. (2019) in their scoping review across 27 primary research studies also explored patient‐provider interactions with patients with autism, but across healthcare settings. The overriding message from this review highlighted the challenges faced by HCPs to enhance communication, collaboration and subsequent training needs. However, needs and outcomes are only relative to participants that have infrequent contact with patients with autism, with only 3 of the 27 included studies of health care providers with specialised training and experience in autism. This suggests that although effective and meaningful patient‐provider communication is challenging for HCPs, it is an advanced clinical practice feature (Skills for Health and Health Education England 2020), and one from this review was not necessarily aligned to HCPs with specialist experience. Furthermore, the review suggests an advanced skill set to work effectively with autistic people is still required by HCPs.

Luzi et al. (2018) in a European project explored interprofessional collaboration across primary care, secondary care and social care professionals to develop personalised plans of care for children with ASD. This demands advanced practitioner capability to analyse and synthesise complex problems not only across different sets of professionals responsible for children with ASD and ADHD, but also different health and social care sectors (HEE 2017). Questionnaire results showed that in half of the countries analysed, a low level of collaboration is reported in implementation of personalised written plans of care. The exception being Ireland and the UK, wherein disability networks provided a focus for integrated care, where the use and access to electronic records facilitate the sharing process.

### 5.4 LEADERSHIP—Interprofessional Collaborative Working

Evidence exploring leadership used exploratory primary studies, including hermeneutic phenomenological inquiry (Arrey et al. 2019), qualitative interviews (Graham et al. 2020; Bonfim et al. 2023), a Delphi study to generate consensus views (O'Hagan et al. 2023). All these studies used and reported established methods, such as hermeneutic data analysis (Arrey et al. 2019), 3‐round Delphi technique (O'Hagan et al. 2023). The ethnographic review by Haines and Brown 2018, was comprehensive in adoption of methods, including use of Noblit and Hare 7‐stage process and critical reflection to enhance reflexivity.

In a meta‐ethnography study, Haines and Brown (2018), in the UK, explored the meaning of HCPs ‘working effectively’ with support workers to improve care. Drawing on an international evidence‐base from 2015, their review highlighted the agility required by HCPs when leading care provision for adults with intellectual abilities. Haines and Brown (2018) highlighted that being able to share knowledge, be adaptable and formulating clear solutions is what is required for support workers to offer effective support. This also highlights an important Leadership role for advanced practitioners in their supervision for support workers, to maintain standards in care (HEE 2017). Similarly, Arrey et al. (2019), also in the UK, explored ‘collaboration’ when intellectual disability nurses work in partnership with palliative care professionals in palliative care settings, responding to the distress of people with communication difficulties. Haines and Brown (2018) and Arrey et al. (2019) showed the ability to build positive and effective working relationships is at the cornerstone of improving care in this area of practice. Showing humane leadership was seen as fundamental for confident and high‐quality care and reflects not only a core aspect of Intellectual Disability practice but also the requirement for ‘compassionate’ leadership as implicit advanced practitioner capabilities (Skills for Health and Health Education England 2020). Knowledge of a culturally acceptable care environment and having ‘experience’ of being involved in the collaboration were all important indicators of good practice and advancing practice in this specialist area of collaboration. Moreover, O'Hagan et al. (2023) in their US based Delphi study, supported this position recommending pragmatic interventions to make autism care ‘more friendly’, such as ensuring a facilitative environmental and staff training to support autistic patients.

Graham et al. (2020) evaluated the role of the pharmacist independent prescriber in a UK community mental health team for intellectual disabilities. HCPs were interviewed to consider the nature of collaborative working and impact on the provision of care. This study highlighted the challenges of the pharmacist independent prescriber fitting in with the organisational hierarchy, often dominated by medical practitioners. From an HCP perspective, there was support for the pharmacist role, although, in this study, cited interview quotes on working with the wider HCPs team (Nurses, psychologist) were limited in number. Bonfim et al. (2023) explored the different types of care offered and the impact of a Family‐Centred Care philosophical framework from three multidisciplinary teams in Brazil; 22 HCPs participated in the study. This study did highlight the many challenges of using a Family Centred model of care. In terms of collaboration, this paper focused more on individual experiences of participants working in different parts of the service, but did indicate nurses could be seen as a positive link to promote collaboration in the MDT, demonstrating the necessary collaborative leadership feature of advanced practice (HEE 2017).

### 5.5 EDUCATION—Knowledge, Attitude and Competence

Evidence exploring education included seven primary studies and two literature reviews. Primary studies explored knowledge and attitude through questionnaires (Melville et al. 2005; Lewis and Stenfert‐Kroese 2010; Bekkema et al. 2014; Mohan and Tiwari 2023; Alaghband Rad et al. 2022), or qualitatively (Blaine et al. 2023; Babb et al. 2021). Good practice in survey design was reported, in validated scales of measurement and use of theoretical frameworks to guide the measurement of attitude; survey response reporting, for example from targeted national surveys, was high (Bekkema et al. 2014). Qualitative approaches also used a theoretical framework, the theoretical domains framework to guide exploration of attitude, showing good levels of content validity and large qualitative samples using validated interview scales (Babb et al. 2021). Both reviews used established review processes and reporting, but Ee et al. (2021) review methodology was not stated.

HCPs in this discipline possess varying degrees of knowledge and competence when practicing in hospital, community and rural environments. Melville et al. (2005), in the UK, investigated practice nurses' attitudes, knowledge, training needs and self‐efficacy in their work with people with an Intellectual Disability. Practice Nurses operate at an enhanced level of practice as experienced nurses with a post graduate qualification (Colivicchi and Evans 2024). By questionnaire, *n* = 201 participants reported positive attitudes and self‐efficacy. However, 86% of participants did report experiencing specific ‘difficulties’ during appointments; and only 8% of participants receiving any training in communicating with people with Intellectual Disability, highlighting a clear gap between skills and training need. Similarly, Lewis and Stenfert‐Kroese (2010) in their questionnaire for hospital‐based staff in the UK, explored emotional reactions towards patients with an intellectual disability. Again, difficulties and negative emotions and fewer positive emotions were reported, in response for caring for a patient with an intellectual disability compared to a patient with a physical disability were reported. Blaine et al. (2023) using semi‐structured phone interviews in a study from California, USA, identified differences between HCPs and parents in perceptions of good care and nutrition. Health professionals acknowledged that their lack of knowledge or capacity to provide nutrition education or referrals was a common barrier to care, particularly given limited community resources.

#### 5.5.1 Knowledge and Training Gaps

Similar challenges in communication were reported by Babb et al. (2021) in their UK qualitative exploration of the experiences of autistic women using eating disorder services. Although much of the data related to patients' experiences, HCPs' views also acknowledge deficits in personal communication and reflect the challenges associated with maintaining advanced level practice requirements with up‐to‐date patient knowledge and competence (HEE 2017). Bekkema et al. (2014) from their survey from *n* = 181 Nurses and Social Workers in the Netherlands reported that most respondents felt inadequately trained in end‐of‐life care issues. Despite a positive appraisal of the end‐of‐life care process, both nurses and social workers felt they could offer better care.

When the focus is on specialist services, knowledge, attitude and confidence are reported differently. Urbanowicz et al. (2020) captured experiences and views of Australian HCPs caring for adults on the autistic spectrum from disability‐specific settings. In this study, self‐perceived knowledge among HCPs was proportionally quite high; 63% felt ‘knowledgeable’ and 62% competent to deliver care. HCPs directly working in specialist service settings felt more confident, demonstrating a knowledge difference to HCPs not working in specialist services who required more direct and supported education. Mohan and Tiwari (2023) from India reinforced the importance of ‘experience’, with HCPs with at least 3 years specialist experience reporting confidence in delivering and enhancing literacy interventions, an observable feature associated with interventions with this group of people (Skills for Health and Health Education England 2020). In their survey, most HCPs (78.5%) cited effective interventions to enhance emergent literary skills in children. These studies highlight the benefits of working alongside and caring for people with intellectual disability and/or autism, which builds confidence in HCPs' capabilities and advanced practice. Moreover, the ongoing professional and autonomous feature of advanced practice requires a continual process of self‐education, ensuring the delivery of competent and knowledgeable care (HEE 2017).

However, Ee et al. (2021) in their systematic review highlighted a clear lack of confidence and negative views of HCPs towards caring for people with intellectual disabilities with mental health problems. In their review, they stressed the need for formalised education, and if not available an effective referral to specialist mental health practitioners. Alaghband Rad et al. (2022) reported similar, concerning findings in their survey of 182 HCPs from Iran. No comparable capabilities framework such as the Multi‐professional capabilities framework (HEE 2017) or registration of HCPs specifically for People with intellectual disability and/or autism exists in Iran. Whilst medical practitioners will diagnose, special educational school provision is in existence (Samadi and McConkey 2011), cultural differences between, for example, the UK, with Iran viewing diagnosis of disability as stigmatising (Samadi 2008). Without sufficient guidelines, a large proportion (67%) reported patients being uncooperative for medical procedures, with a clear need for training on communication strategies to enhance care. Finally, Corden et al. (2022) systematic review captured collective HCPs' views on knowledge, self‐efficacy and attitudes towards working with autistic people in their systematic review from *n* = 35 studies. Overall, across studies, indicated low to moderate levels of knowledge and perceived self‐efficacy about autism among health workers across a range of backgrounds and countries; not explained by demographic or professional background differences.

Collectively these studies highlight challenges and threats to good practice in caring for those with an intellectual disability and/or autism. Education and practice leadership (HEE 2017) is the driver that can help resolve these challenges.

### 5.6 PRACTICE DEVELOPMENT AND RESEARCH—Role Evaluation, Models and Tools

Evidence exploring practice development used a variety of primary research methods, including mixed methods, survey and qualitative approaches. Again, good examples of methodological rigour (CASP, n.d.) are reported, for example, large and comprehensive qualitative sampling (Brown et al. 2021); questionnaire reliability checks (Oulton et al. 2019); validated questionnaire items through previous focus groups (Doody et al. 2019); purposive sampling to add a rich perspectives from diverse and targeted sample (Ní Riain and Wickham 2023) and a variety of inclusive sampling to develop screening tools (Squires et al. 1996). Both reviews used and executed established integrative review methodology (Whittemore and Knafl 2005) and PRIMSA to guide reporting (Jaques et al. 2018).

Role evaluation was an important feature of practice development. Brown et al. (2012) in their UK mixed methods study, evaluated the role of Intellectual Disability Liaison Nursing services as specialist support for HCPs, in primary care and secondary care. From analysis of *n* = 323 referrals, reported a positive impact on clinical care, education and practice strategic organisational developments; with a model of care being developed to support patients with an intellectual disability to achieve health outcomes when attending general hospitals. Similarly, with a focus on attitudes, perceptions and opinions, Doody et al. (2017a, 2017b) explored the role of clinical nurse specialists (CNS) in Intellectual Disability Nursing in Ireland. Similar to practices identified by Brown et al. (2012), intellectual disability clinical nurse specialists contribute to and support care delivery across a range of areas. Offering a nuanced account of impact, this large qualitative study highlighted how using specialist knowledge advances practice and has a positive impact on care delivery. Further, Doody et al. (2019) canvassing views from the MDT of the role of Clinical Nurse Specialists in Ireland reported the significance and contribution and the support they provide for all stakeholders within the care process, illuminating the key feature of collaborative leadership and clinical practice (Skills for Health and Health Education England 2020). Despite the complexity of the role, HCPs colleagues recognised areas in which specialists do advance practice across many services, in particular showing leadership in inter‐agency collaboration (HEE 2017). However, from an international perspective, this survey is confined by sampling from eight intellectual disability sites in Ireland.

In 2022, Doody et al. (2023) updated the evidence on Clinical Nurse Specialists (CNS) in their integrative review, accessing international literature. Similar positive attributes from previous studies (Brown et al. 2012; Doody et al. 2017a, 2017b) were reported, framed as the biopsychosocial‐educational approach to addressing the physical, mental and social needs of clients with intellectual disability. However, Doody et al. (2023) did highlight scope to advance practice, identifying limited literature on the assessment and intervention strategies employed by intellectual disability nurses, presenting a gap in the advanced practice portfolio for Intellectual Disability Nurses.

#### 5.6.1 Advanced Practice‐Communication Skills

Jaques et al. (2018) in their integrative review of the intellectual disability nurse explored their specific skills. Similar to outcomes generated by Brown et al. (2012) and Doody et al. (2017a, 2017b), synthesis identified the specialist communication skills as being uniquely relational and the need to be patient and resilient to enhance communication. This is a core feature of advancing practice for people with intellectual disability and/or autism (Skills for Health and Health Education England 2020) and marks out its professional care contribution compared to other fields of practice (Brown et al. 2012, Doody et al. 2017a, 2017b).

Oulton et al. (2019) provided further insight into the value and impact of dedicated intellectual disability nurse care provision across 15 Children's hospitals in England, exploring the impact on quality, standards of care and influence on other HCPs confidence to carry out care. From a largescale survey (*n* = 1681) and interview data (*n* = 47 participants) reported that this role as being highly valued by hospital staff; but did not show an increase in staff perceptions of their capacity, or confidence. Therefore, highlighting the need to develop advanced practice particularly in leadership and education of intellectual disability nurses (Skills for Health and Health Education England 2020), to further enable advanced practitioners to have an educational influence on HCP who are working in different settings. Ní Riain and Wickham (2023) in Ireland, further added to the evidence based in ‘role evaluation’ for advanced practice, by exploring the role in an intellectual disability community organisation, rather than in hospitals. The findings from this qualitative study were similar to previous studies (Brown et al. 2012; Doody et al. 2017a, 2017b), which demonstrated the competence required for advanced practice in a community setting and particularly standard setting in education.

A feature of advanced practice for People with intellectual disability and/or autism is the development and implementation of screening (Lloyd and Coulson 2014) and assessment tools development (Skills for Health and Health Education England 2020). This review however retrieved very few studies having developed tools/checklists or models of care. Squires et al. (1996) development of a screening model for pre‐school age children validated their tool using parent and HCP validation. Squires et al. (1996), in the USA, focused on preschool‐age children with suspected delays in cognitive, motor, language and behavioural ages. It was suggested that health care professionals and physicians use developmental tests that have established psychometric properties and are culturally sensitive and comprehensive. Simpson (2020) in the UK, also focused on tool developmental, to enhance an Autism‐Friendly environment for various health care settings. Piloted across several clinical settings and endorsed by NICE (2020) recommendations included reducing loud noises and clear signage to help avoid patient anxiety and asking patients if the environment was comfortable. All usable indicators of impact to enhance the environment. The development and implementation of these screening tools across different settings requires advanced level evidenced informed practice capability (HEE 2017) and is beyond the standard scope of Intellectual disability practice. Finally, on a specialist autism services' ward in the UK, Rowe and Nevin (2013) in their service improvement pilot project, explored the potential to bring in ‘patient voice’ into care planning. From *n* = 4 case studies of patients with mild–severe autism, overseen by a psychologist, person‐centred communication was enhanced. Higher expertise is needed with patients with severe symptoms and is a key clinical aspect of advanced practice development when skilfully involving individuals in their care planning (Skills for Health and Health Education England 2020).

In summary, across all themes the key message is that HCPs' efforts to develop advanced practice to improve care have been applied across many different HCPs working in many different clinical settings. Working in an area of complex care provision, the review indicates HCPs provide nuanced practice contributions for example mental health assessments, connected leadership examples across multiple pathways, distinctive work educating others and research and innovation activity improving services for People with Intellectual Disability or Autism.

## 6 Discussion

The review had three aims: to investigate the context and development of advanced practice (journey), to identify the positionality of advanced practice in intellectual disability and autism, and thirdly to explore the impact of advanced practice for People with intellectual disability and/or autism. Through the lens of the multi‐professional capabilities for advanced practice (HEE 2017).

### 6.1 Context and Development

Working with People with intellectual disability and/or autism at an advanced level centres on the capacity to build effective relationships and deliver responsive person‐centred interventions (Brown et al. 2012). Several UK papers depict this context and demonstrate how care can be progressed by working more effectively in partnership with patients and service users through practice developments, primarily by Intellectual Disability Nurses and AHPs (MacArthur et al. 2015; Morris et al. 2019; Doody et al. 2019). CNSs in Ireland have expanded their role showing the contribution they make for all stakeholders and the care process (Doody et al. 2017a, 2017b; 2018). This care contribution resonates with a study in Scotland through the role of Acute Liaison Nurse (Brown et al. 2012). This nuanced model of care highlights key areas of advanced practice, such as collaborating, educating and strategically influencing health care professionals working in acute care. This area of advanced practice is important as clinicians working in acute care have reported uncertainty when working with this population group (Desroches et al. 2022). Advanced practitioners in this field can advise acute care staff on person centred clinical safety guidelines and best interest decision making, ensuring clinicians operate within legal frameworks (MacArthur et al. 2015). Ní Riain and Wickham (2023) adds to role evaluation in developing the practice evidence‐base, with a focus on the community perspectives of acute hospital care and additionally evident in relational capacity in forensic settings (Lovell and Skellern 2020) and Doody et al. (2022), via a bio‐psycho‐social educational approach meeting the diverse needs of people with Intellectual Disability and/or Autism.

A picture emerges of the journey of advanced practice development for People with intellectual disability and/or autism, as illustrated by health management, education of others and health promotion, whilst also acknowledging knowledge deficits in assessment and intervention strategies (Doody et al. 2022). Whilst these areas are synonymous with the policy trajectory of community and primary care prevention within the NHS Long Term Plan (2024) in England, more training is required to further enhance assessment and intervention skills at an advanced practice level.

Studies from the Netherlands, Noorlandt et al. (2020) and Bekkema et al. (2014) focused on end‐of‐life care. Respondents felt inadequately trained in end‐of‐life care practice (Bekkema et al. 2014) and end‐of‐life decision making (Noorlandt et al. 2020). Burke et al. (2016) revealed the complexities of assessing mental health conditions in children with autism and the requirement for more education to be able to manage care effectively. Similarly, Pridding and Procter (2008) in Australia found NPs were challenged by assessing personality disorder in people with intellectual disability and/or autism. Morris et al. (2019) indicated positive attitudes and self‐efficacy towards AHPs' work with people with intellectual disability and/or autism; however, practice unease and uncertainty were associated with different diagnostic interpretations and complexities that people with autism present with. The multi‐dimensional challenges of assessing and responding effectively to people with autism and gender dysphoria also indicated a further requirement for advanced practice development (Gagnon et al. 2023).

Evidence of innovative models of practice with children with and without autism were evident and represent commonalities across how HCPs are developing advanced practice care. Development of screening models; strength‐based Occupational Therapy (OT) interventions with positive mental health outcomes, OT led mealtime interventions, high technological environments, literacy interventions, belief barriers and enablers for nutritional care and family centred care (Squires et al. 1996; Murthi et al. 2023; Pettersson et al. 2023; Barrientos et al. 2023, Mohan and Tiwari 2023; Blaine et al. 2023; Bonfim et al. 2023). Innovative practice developments were also evident, such as the pharmacist independent prescriber which further evidenced the positive impact and influence HCPs can have in the care journey for People with intellectual disability and/or autism (Graham et al. 2020) as well as via environment friendly autism tools (Simpson 2020), and responsive approaches to the needs of autistic women in emergency departments (Babb et al. 2021). Greater Knowledge of the innovation and nuances associated with advanced practice in this field could be invested more, at a post‐graduate advanced level of training.

### 6.2 Positionality (PILLARS of Advancing Practice)

In terms of Clinical Practice, the UK literature was mainly advancing care alongside roles such as CNS; however, international literature reveals evidence of innovation specifically with children. Within the Leadership and Management pillar, HCPs demonstrated leadership in inter‐professional cross‐sector working and in relational, person‐centred domains of practice. An emphasis was placed on the pillar of Education. Knowledge gaps were evident for both AHPs working with people with intellectual disability and/or autism and other professionals at the point of contact with people with intellectual disability and/or autism. Evidence‐based practice centred critically on the development of assessment and screening tools and the impact of advanced practitioners working in different settings. Although there are very limited advanced practice educational programmes in existence, some PG advanced programmes in this field have been able to develop more confident and evidence‐informed practitioners on completion (Bollard et al. 2025). The breadth, complexity and positionality of advanced practice this review has illustrated, points to the potential emergence of new pillars, such as technological advances/artificial intelligence and citizenship and disadvantaged community development.

### 6.3 Limitations

Scoping review methodologies have evolved and embraced a rigorous and systematic process of similar reviews, such as integrative review. Applying a step‐like process and using PRISMA‐ScR to guide reporting enhanced validity and reliability. Due to a reported cyber‐attack, it was not possible to retrieve grey literature from the British Library; this said, the database search strategy was sufficiently sensitive to retrieve some grey literature, for example from the WOS database. A fuller inclusion of grey literature from policy and theory driven papers could have enhanced professional perspectives and potential themes; hence the scope of this review being limited to primary and secondary empirical evidence. The scope of literature could have been enhanced by English Language studies, but translated papers were included. Interpretation and evaluation of data intentions are to avoid bias, but there is always an element of subjectivity and multiple perspectives of the review team which could be seen as a limitation. Furthermore, this review did not produce a formal ‘quality evaluation of included papers’ but did present a clear picture of the type of evidence reviewed, including observations on methodological quality in the review of results of the scoping review. In this way, the quality of studies was considered alongside synthesised outcomes.

## 7 Conclusion

Providing effective advanced practice care for people with intellectual disability and/or autism requires a high level of skill, knowledge and experience exercised across different roles and settings. The complexity and neurodiverse needs of this population group present practice challenges to both those working in specialised and non‐specialised provisions of care. Like other areas of advancing practice, there is no universal practice model to assist empirical international role evaluation.

Variable terminology is adopted in different counties to describe and categorise this population group. In addition to this, varied international health and social care configurations exist and may go some way to explain why some countries have invested in the role in a more structured way than other countries. The multi‐professional capabilities framework (HEE 2017) and domains of care (Skills for Health and Health Education England 2020) *when working with people who have an intellectual disability and/or autism*, offer a useful standardised mechanism to construct domains of advanced practice and are enabling advanced practitioner role development in England. Other nations, such as in Ireland and Northern Ireland, provide exemplars of advancing practice in this field. In line with the trajectory of health care delivery in the UK and globally, new pillars such as how advanced practice adopts technological advances/artificial intelligence and promotes citizenship and disadvantaged community development, could capture the wider nuances of advanced practice further. In the absence of international advanced practitioner role comparators, more research and workforce development are required to standardise and expand a professional framework, inclusive of advanced practice for this group of people. This is of timely importance in England particularly. Recent applicants choosing to undertake this field of practice in England have declined, and more than 40% of universities have closed their RNLD programmes (Council of Deans 2023). Demonstrating the value and contribution of advanced practice and what this career destination point might look like could attract young people to the profession. While the lack of a universal practice model makes the development of this challenging, a possible starting point would be to ask people with intellectual disability and/or autism themselves to identify specific domains of advanced practice that would enhance their lives.

Most of the evidence base supporting the findings of this review is of sound methodological quality, with established methods reported and executed across studies. In the journey towards advancing practice, there is a clear professional and practice motivation to explore and develop practice, evidenced through themes developed in this review. The theme ‘practice development and role evaluation’ demonstrates a more focused approach to theory building through empirical investigation; offering an insight into the impact of advancing practice activities, with much of this empirical investigation being UK based. Further empirical investigation, using exploratory methods, should aim to build on this review's findings, identified as an international reference point for advancing practice.

### 7.1 Recommendations

Next steps are summarised:

- An international advanced practice summit galvanising nominated international practice jurisdictions may assist in helping to coordinate a timely workforce development response. A cross‐UK consensus approach, as a policy initiative to develop advanced practice and workforce in intellectual disability care.
- A national and cross‐UK workforce re‐emphasis on the difference advanced practice programmes make both to service delivery and for people with intellectual disability and/or autism.
- Commissioning of new training options for both the development of advanced clinical practitioners and the training these practitioners offer to others, with consideration of 2 new pillars of practice, namely Technological advances/AI and Citizenship and Disadvantaged community development
- International recognition of the available opportunities to develop the role of advancing practice, given the current empirical progress in understanding ‘assessing and managing care’, ‘working in partnership’, ‘Interprofessional collaboration’, ‘Knowledge and attitude’ and ‘practice development’, and importantly the decline in this professional grouping in England at a pre‐registration level and lack of PG advanced practice programmes

## Funding

This work was supported by NHSE.

## Conflicts of Interest

The authors declare no conflicts of interest.

## Supporting information


**Table S1:** Data extraction table.

## Data Availability

The data that support the findings of this study are available from the corresponding author upon reasonable request.

## References

[jocn70494-bib-0002] Al Sharif, S. , and S. Ratnapalan . 2016. “Managing Children With Autism Spectrum Disorders in Emergency Departments.” Pediatric Emergency Care 32, no. 2: 101–103. 10.1097/PEC.0000000000000705. 26835567

[jocn70494-bib-0003] Alaghband Rad, J. , M. Ardeshir , E. Hakki Kkazazi , and M. Mtamed . 2022. “Adult Autism Spectrum Disorder: Knowledge, Attitude and Practice of Health System Professionals.” Journal of Mental Health Training 18, no. 3: 185–194. 10.1108/JMHTEP-07-2022-0066.

[jocn70494-bib-0004] Arksey, H. , and L. O'Malley . 2005. “Scoping Studies: Towards a Methodological Framework.” International Journal of Social Research Methodology: Theory & Practice 8, no. 1: 19–32.

[jocn70494-bib-0005] Arrey, S. K. , M. N. Kirshbaum , and V. Finn . 2019. “In Search of Care Strategies for Distressed People With Communication Difficulties and a Learning Disability in Palliative Care Settings: The Lived Experiences of Registered Learning Disability Nurses and Palliative Care Professionals.” Journal of Research in Nursing 24, no. 6: 386–400. 10.1177/1744987118764532. PMC7932262 34394552

[jocn70494-bib-0006] Auberry, K. , and D. Cullen . 2016. “Implementation of an Evidence‐Based Seizure Algorithm in Intellectual Disability Nursing.” Journal of Intellectual Disabilities 20, no. 1: 55–64. 10.1177/1744629515598216. 26283660

[jocn70494-bib-0007] Aveyard, H. 2018. Doing a Literature Review in Health and Social Care: A Practical Guide. 5th ed. Open University Press.

[jocn70494-bib-0008] Babb, C. , J. Brede , R. G. J. Catherine , et al. 2021. “ *It's Not That They Don't Want to Access the Support … It's the Impact of the Autism*: The Experience of Eating Disorder Services From the Perspective of Autistic Women, Parents and Healthcare Professionals.” Autism 25, no. 5: 1409–1421. 10.1177/1362361321991257. PMC8264634 33588579

[jocn70494-bib-0009] Barrientos, B. V. , A. T. Badajos , E. M. Bucog , R. C. Mauro , and P. P. Bulan . 2023. “Mealtime Experiences of Children With Autism Spectrum Disorder From the Perspectives of Filipino Occupational Therapists in Cebu: Implications for Practice.” Internet Journal of Allied Health Sciences and Practice 21, no. 1: 2.

[jocn70494-bib-0010] Bekkema, N. , A. J. E. de Veer , G. Albers , C. M. P. M. Hertogh , B. D. Onwuteaka‐Philipsen , and A. L. Francke . 2014. “Training Needs of Nurses and Social Workers in the End‐Of‐Life Care for People With Intellectual Disabilities: A National Survey.” Nurse Education Today 34, no. 4: 494–500. 10.1016/j.nedt.2013.07.018. 23972684

[jocn70494-bib-0091] Biswas, A. B. , P. Fletcher , G. Hurst , M. Sweeney , and K. Taylor . 2010. “Obesity in People With Learning Disabilities: Possible Causes and Reduction Interventions.” Nursing Times 106, no. 14: 16–18. 20806643

[jocn70494-bib-0011] Blaine, R. E. , K. P. Blaine , K. Cheng , C. Banuelos , and A. Leal . 2023. “Priorities, Barriers, and Facilitators for Nutrition‐Related Care for Autistic Children: A Qualitative Study Comparing Interdisciplinary Health Professional and Parent Perspectives.” Frontiers in Pediatrics 11: 1198177. 10.3389/fped.2023.1198177. PMC10465129 37650046

[jocn70494-bib-0012] Bollard, M. , B. Appleby , and S. Kempson . 2025. “Investigating Advanced Practice for People With Intellectual Disability and Autism: A Mixed Methods Study.” Journal of Clinical Nursing 34: 3146–3159. 10.1111/jocn.17696. 40474486

[jocn70494-bib-0013] Bollard, M. , and M. J. D. Jukes . 1999. “Specialist Practitioner Within Community Learning Disabilities and the Primary Health Care Team.” Journal of Learning Disabilities 3, no. 1: 11–19.

[jocn70494-bib-0014] Bonfim, T. D. A. , B. C. C. Giacon‐Arruda , S. A. F. Galera , E. F. Teston , F. G. P. D. Nascimento , and M. A. Marcheti . 2023. Assistance to Families of Children With Autism Spectrum Disorders: Perceptions of the Multiprofessional Team. FapUNIFESP (SciELO). 10.1590/1518-8345.5694.3780 PMC9990997 36888790

[jocn70494-bib-0015] Booth, A. 2009. “Unpacking Your Literature Search Toolbox: On Search Styles and Tactics.” Health Information and Libraries Journal 25: 313–317. 10.1111/j.1471-1842.2008.00825. 19076679

[jocn70494-bib-0095] British Library . 2024. “Learning Lessons From the Cyber‐Attack.” *British Library*, 8 March, 2024. https://www.bl.uk/storeis/blogs/posts/learning‐lessons‐from‐the‐cyber‐attcak.

[jocn70494-bib-0016] Brown, M. , J. MacArthur , A. McKechanie , S. Mack , M. Hayes , and J. Fletcher . 2012. “Learning Disability Liaison Nursing Services in South‐East Scotland: A Mixed‐Methods Impact and Outcome Study.” Journal of Intellectual Disability Research 56, no. 12: 1161–1174. 10.1111/j.1365-2788.2011.01511.x. 22142456

[jocn70494-bib-0098] Brown, M. , J. MacArthur , M. Truesdale , and A. Higgins . 2021. “The Transition From Child to Adult Health Services for Young Adults With Intellectual Disabilities: An Evaluation of a Pilot of an Online Learning Resource for Registered Nurses.” Nurse Education in Practice 64: 10342. 10.1016/j.nepr.2022.103424 35947941

[jocn70494-bib-0017] Burke, D. A. , H. M. Koot , A. de Wilde , and S. Begeer . 2016. “Influence of Child Factors on Health‐Care Professionals' Recognition of Common Childhood Mental‐Health Problems.” Journal of Child and Family Studies 25, no. 10: 3083–3096. 10.1007/s10826-016-0475-9. PMC5016556 27656089

[jocn70494-bib-0018] Canadian Nurses Association . 2019. Advanced Practice Nursing: A Pan Canadian Framework. CAN.

[jocn70494-bib-0019] Christmals, C. D. , and G. J. Gross . 2017. “An Integrative Literature Review Framework for Postgraduate Nursing Research Reviews.” European Journal of Research in Medical Sciences 5, no. 1: 7–15.

[jocn70494-bib-0020] Colivicchi, A. , and R. Evans . 2024. “Enhanced Practice Nurses To Be Included in ARRS Under New Contract.” Management in Practice. https://managementinpractice.com/news/enhanced‐practice‐nurses‐to‐be‐included‐in‐arrs‐under‐new‐contract/.

[jocn70494-bib-0021] Cooke, A. , D. Smith , and A. Booth . 2012. “Beyond PICO: The SPIDER Tool for Qualitative Evidence Synthesis.” Qualitative Health Research 22, no. 10: 1435–1443. 10.1177/1049732312452938 22829486

[jocn70494-bib-0022] Cooper, S. A. , L. Hughes‐McCormack , N. Greenlaw , et al. 2018. “Management and Prevalence of Long‐Term Conditions in Primary Health Care for Adults With Intellectual Disabilities Compared With the General Population: A Population‐Based Cohort Study.” Journal of Applied Research in Intellectual Disabilities 31, no. S.1: 68–81. 10.1111/jar.12386 28730746

[jocn70494-bib-0023] Corden, K. , R. Brewer , and E. Cage . 2022. “A Systematic Review of Healthcare Professionals' Knowledge, Self‐Efficacy and Attitudes Towards Working With Autistic People.” Review Journal of Autism and Developmental Disorders 9, no. 3: 386–399.

[jocn70494-bib-0100] Council of Deans of Health . 2023. Learning Disability Nursing: Programme Sustainability Report . https://councilofdeans.org.uk.

[jocn70494-bib-0024] Critical Appraisal Skills Programme (CASP) . n.d. “CASP Checklists.” https://casp‐uk.net/casp‐tools‐checklists/.

[jocn70494-bib-0025] Dalgarno, M. F. , and S. A. Riordan . 2014. “Forensic Intellectual Disability Nursing: What's It Really Like?” Journal of Intellectual Disabilities and Offending Behaviour 5, no. 4: 167–177. 10.1108/JIDOB-11-2014-0017.

[jocn70494-bib-0026] Daniels, N. F. , B. Coughlan , and R. Duschinsky . 2021. “How UK Health Care Professionals Conceptualise Parental Experiences of the Diagnostic Process for Autism Spectrum Disorder: A Qualitative Study.” SAGE Open Medicine 9: 20503121211031310. 10.1177/20503121211031310. PMC8287381 34349995

[jocn70494-bib-0027] Desroches, M. L. , V. A. Howie , N. J. Wilson , and P. Lewis . 2022. “Nurses' Attitudes and Emotions Toward Caring for Adults With Intellectual Disability: An International Replication Study.” Journal of Nursing Scholarship 54, no. 1: 117–124. 10.1111/jnu.12713. 34741386

[jocn70494-bib-0028] Dixon‐Woods, M. , S. Agarwall , B. Young , D. Jones , and A. Sutton . 2004. Integrative Approaches to Qualitative and Quantitative Evidence. Health Development Agency. NHS. https://www.hda.nhs.uk.

[jocn70494-bib-0099] Doody, O. , T. Hennessy , and A. M. Bright . 2022. “The Role and Key Activities of Clinical Nurse Specialists and Advanced Nurse Practitioners in Supporting Healthcare Provision for People With Intellectual Disability: An Integrative Review.” International Journal of Nursing Studies 129: 104207. 10.1016/j.ijnurstu.2022.104207 35349972

[jocn70494-bib-0029] Doody, O. , T. Hennessy , M. Moloney , R. Lyons , and A. M. Bright . 2023. “The Value and Contribution of Intellectual Disability Nurses/Nurses Caring for People With Intellectual Disability in Intellectual Disability Settings: A Scoping Review.” Journal of Clinical Nursing 32, no. 9–10: 1993–2040. 10.1111/jocn.16289. 35301775

[jocn70494-bib-0030] Doody, O. , R. Lyons , and R. Ryan . 2019. “The Experiences of Adults With Intellectual Disability in the Involvement of Nursing Care Planning in Health Services.” British Journal of Learning Disabilities 47, no. 4: 233–240. 10.1111/bld.12281.

[jocn70494-bib-0031] Doody, O. , E. Slevin , and L. Taggart . 2017a. “Activities of Intellectual Disability Clinical Nurse Specialists in Ireland.” Clinical Nurse Specialist 31, no. 2: 89–96. 10.1097/NUR.0000000000000278. 28169896

[jocn70494-bib-0032] Doody, O. , E. Slevin , and L. Taggart . 2017b. “Focus Group Interviews Examining the Contribution of Intellectual Disability Clinical Nurse Specialists in Ireland.” Journal of Clinical Nursing 26, no. 19–20: 2964–2975. 10.1111/jocn.13636. 27862506

[jocn70494-bib-0033] Ee, J. , B. Stenfert Kroese , and J. Rose . 2021. “A Systematic Review of the Knowledge, Attitudes and Perceptions of Health and Social Care Professionals Towards People With Learning Disabilities and Mental Health Problems.” British Journal of Learning Disabilities 50, no. 4: 467–483. 10.1111/bld.12401.

[jocn70494-bib-0034] Evans, D. 2023. Making Sense of Evidence‐Based Practice for Nursing. An Introduction to Quantitative and Qualitative Research and Systematic Reviews. Routledge.

[jocn70494-bib-0035] Finfgeld‐Connett, D. 2018. A Guide to Qualitative Meta‐Synthesis. Taylor Francis.

[jocn70494-bib-0036] Fong Yeong Woo, B. , Z. Wentao , W. L. Toon , and W. S. S. Tam . 2019. “Practice Patterns and Role Perception of Advanced Practice Nurses: A Nationwide Cross‐Sectional *Study* .” Journal of Nursing Management 29: 992–1004. 10.1111/jonm.12759 30776163

[jocn70494-bib-0037] Gagnon, D. , V. Pellerin , É. Trudel , A. Vincent , and E. Morales . 2023. “ASD and Gender Dysphoria: A Review of Challenges and Facilitators for Health Professionals.” Sexuality and Disability 41, no. 3: 691–706. 10.1007/s11195-023-09791-1.

[jocn70494-bib-0038] Gardner, G. , C. Duffield , and A. Gardner . 2017. The Australian Advanced Practice Nursing Self‐Appraisal Tool (The ADVANCE Tool). Queensland University of Technology.

[jocn70494-bib-0039] Glover, G. , R. Williams , P. Heslop , J. Oyinlola , and J. Grey . 2017. “Mortality in People With Intellectual Disabilities in England.” Journal of Intellectual Disability Research 61, no. 1: 62–74. 10.1111/jir.12314 27489075

[jocn70494-bib-0040] Graham, Y. N. H. , D. Gerrard , S. Laight , R. Brown , S. Keith , and C. Hayes . 2020. “More Than Medication: Evaluating the Role of the Pharmacist Independent Prescriber in a Community Team for Learning Disabilities and Behaviours Deemed To Be Challenging.” British Journal of Learning Disabilities 48, no. 3: 232–241. 10.1111/bld.12323.

[jocn70494-bib-0041] Graneheim, U. H. , and B. Lundman . 2003. “Qualitative Content Analysis in Nursing Research: Concepts, Procedures and Measures to Achieve Trustworthiness.” Nurse Education Today 24: 105–112. 10.1016/j.nedt.2003.10.001 14769454

[jocn70494-bib-0042] Haines, D. , and A. Brown . 2018. “Health Professionals Working Effectively With Support Workers to Enhance the Quality of Support for Adults With Intellectual Disabilities: A Meta‐Ethnography.” Journal of Applied Research in Intellectual Disabilities 31, no. 2: 200–212. 10.1111/jar.12343. 28224690

[jocn70494-bib-0043] Health Education England . 2017. “Multi‐Professional Framework for Advanced Clinical Practice in England.” https://www.hee.nhs.uk/our‐work/advanced‐clinical‐practice/multi‐professionalframework.

[jocn70494-bib-0097] Health Education England . 2019. Multi‐Professional Framework for Advanced Clinical Practice in England. Health Education England.

[jocn70494-bib-0044] Heslop, P. , P. S. Blair , P. Fleming , M. Hoghton , A. Marriott , and L. Russ . 2014. “The Confidential Inquiry Into Premature Deaths of People With Intellectual Disabilities in the UK: A Population‐Based Study.” Lancet 383, no. 9920: 889–895. 10.1016/S0140-6736(13)62026-7 24332307

[jocn70494-bib-0045] Holmes, L. G. , M. B. Himle , K. K. Sewell , P. S. Carbone , D. S. Strass[1]berg , and N. A. Murphy . 2014. “Addressing Sexuality in Youth With Autism Spectrum Disorders: Current Pediatric Practices and Barriers.” Journal of Developmental & Behavioral Pediatrics 35, no. 3: 172–178. 10.1097/DBP.0000000000000030 24651831

[jocn70494-bib-0046] Hosking, F. J. , I. M. Carey , S. M. Shah , et al. 2016. “Mortality Among Adults With Intellectual Disability in England: Comparisons With the General Population.” American Journal of Public Health 106, no. 8: 1483–1490. 10.2105/AJPH.2016.303240. PMC4940652 27310347

[jocn70494-bib-0047] Htay, M. , and D. Whitehead . 2021. “The Effectiveness of the Role of Advanced Nurse Practitioners Compared to Physician‐Led or Usual Care: A Systematic Review.” International Journal of Nursing Studies Advances 3: 100034. 10.1016/j.ijnsa.2021.100034. PMC11080477 38746729

[jocn70494-bib-0048] International Council of Nurses . 2020. Guidelines on Advanced Practice Nursing. ICN.

[jocn70494-bib-0049] Jaques, H. , P. Lewis , K. O'Reilly , M. Wiese , and N. J. Wilson . 2018. Understanding the Contemporary Role of the Intellectual Disability Nurse: A Review of the Literature. Wiley. 10.1111/jocn.14555 29893440

[jocn70494-bib-0050] Johnsson, G. , R. Kerslake , and S. Crook . 2019. “Delivering Allied Health Services to Regional and Remote Participants on the Autism Spectrum via Video‐Conferencing Technology: Lessons Learned.” Rural and Remote Health 19, no. 3: 5358. 10.22605/RRH5358. 31474113

[jocn70494-bib-0051] Jukes, M. J. D. , and M. Bollard . 2003. Contemporary Learning Disability Practice. Quay Books.

[jocn70494-bib-0052] Kestenbaum, B. 2019. “General Considerations in Epidemiological Research.” In Epidemiology and Biostatistics: An Introduction to Clinical Research, 2nd ed. Springer Nature.

[jocn70494-bib-0053] Khalil, H. , M. Peters , C. M. Godfrey , P. McInerney , C. B. Soares , and D. Parker . 2016. “An Evidence‐Based Approach to Scoping Reviews.” Worldviews on Evidence‐Based Nursing 13, no. 2: 118–123. 10.1111/wvn.12144 26821833

[jocn70494-bib-0054] King, R. , A. Tod , and T. Sanders . 2017. “Development and Regulation of Advanced Nurse Practitioners in the UK and Internationally.” Nursing Standard 32, no. 14: 43–50. 10.7748/ns.2017.e10858 29185641

[jocn70494-bib-0055] Laurant, M. , M. van der Biezen , N. Wijers , K. Watananirun , E. Kontopantelis , and A. J. van Vught . 2018. “Nurses as Substitutes for Doctors in Primary Care.” Cochrane Database of Systematic Reviews 7, no. 7: CD001271. 10.1002/14651858. PMC6367893 30011347

[jocn70494-bib-0056] Lewis, S. , and B. Stenfert‐Kroese . 2010. “An Investigation of Nursing Staff Attitudes and Emotional Reactions Towards Patients With Intellectual Disability in a General Hospital Setting.” Journal of Applied Research in Intellectual Disabilities 23, no. 4: 355–365. 10.1111/j.1468-3148.2009.00542.x.

[jocn70494-bib-0057] Lloyd, J. L. , and N. S. Coulson . 2014. “The Role of Learning Disability Nurses in Promoting Cervical Screening Uptake in Women With Intellectual Disabilities.” Journal of Intellectual Disabilities 18, no. 2: 129–145. 10.1177/1744629514528829. 24698957

[jocn70494-bib-0058] Lovell, A. , and J. Skellern . 2020. “Making Sense of Complexity: A Qualitative Investigation Into Forensic Learning Disability Nurses' Interpretation of the Contribution of Personal History to Offending Behaviour.” British Journal of Learning Disabilities 48, no. 3: 242–250.

[jocn70494-bib-0093] Lovell, M. 2021. “Commentary: ‘Learning Disability’ vs ‘Intellectual Disability’ – Why the Terminology Matters.” Child and Adolescent Mental Health 26, no. 4: 317–318.

[jocn70494-bib-0059] Luzi, D. , F. Pecoraro , and O. Tamburis . 2018. “Modelling Levels of Collaboration Among Health and Social Care Professionals in the Management of a Mental Health Plan.” Studies in Health Technology and Informatics 247: 516–520. 10.3233/978-1-61499-852-5-516. 29678014

[jocn70494-bib-0060] MacArthur, J. , M. Brown , A. McKechanie , S. Mack , M. Hayes , and J. Fletcher . 2015. “Making Reasonable and Achievable Adjustments: The Contributions of Learning Disability Liaison Nurses in ‘Getting It Right’ for People With Learning Disabilities Receiving General Hospitals Care.” Journal of Advanced Nursing 71, no. 7: 1552–1563. 10.1111/jan.12629. 25682796

[jocn70494-bib-0061] Mafuba, K. , J. Kiernan , C. Kudita , H. M. Chapman , D. Kupara , and R. Chester . 2023. “Understanding the Contribution of Intellectual Disabilities Nurses: Paper 3 of 4 – Evaluation.” Journal of Intellectual Disabilities 30, no. 2: 222–242. 10.1177/17446295231196588. PMC13230659 37611566

[jocn70494-bib-0062] Mason, T. , and D. Phipps . 2010. “Forensic Learning Disability Nursing Skills and Competencies: A Study of Forensic and Non‐Forensic Nurses.” Issues in Mental Health Nursing 31, no. 11: 708–715. 10.3109/01612840.2010.500793 20936892

[jocn70494-bib-0063] Melville, C. A. , J. Finlayson , S. Cooper , et al. 2005. “Enhancing Primary Health Care Services for Adults With Intellectual Disabilities.” Journal of Intellectual Disability Research 49, no. 3: 190–198. 10.1111/j.1365-2788.2005.00640.x. 15713194

[jocn70494-bib-0064] Miles, M. , and A. Huberman . 2013. Qualitative Data Analysis; a Methods Sourcebook. Sage Publications.

[jocn70494-bib-0065] Mohan, H. S. , and S. Tiwari . 2023. “A Survey on Professionals' Beliefs and Practices Related to Emergent Literacy Skills in Children With Autism Spectrum Disorder.” Speech, Language and Hearing 26: 278–288. 10.1080/2050571X.2023.2226532.

[jocn70494-bib-0066] Morris, R. , A. Greenblatt , and M. Saini . 2019. “Healthcare Providers' Experiences With Autism: A Scoping Review.” Journal of Autism and Developmental Disorders 49, no. 6: 2374–2388. 10.1007/s10803-019-03912-6. 30758692

[jocn70494-bib-0067] Munn, Z. , M. D. J. Peters , C. Stern , C. Tufanaru , A. McArthur , and E. Aromataris . 2018. “Systematic Review or Scoping Review? Guidance for Authors When Choosing Between a Systematic or Scoping Review Approach.” BMC Medical Research Methodology 18, no. 1: 1–7. 10.1186/s12874-018-0611-x PMC6245623 30453902

[jocn70494-bib-0094] Munn, Z. , D. Pollock , H. Khalil , et al. 2022. “What are Scoping Reviews? Providing a Formal Definition of Scoping Reviews as a Type of Evidence Synthesis.” JBI Evidence Synthesis 20, no. 4: 950–952. 10.11124/JBIES-21-00483 35249995

[jocn70494-bib-0068] Murthi, K. , Y. Chen , S. Shore , and K. Patten . 2023. “Strengths‐Based Practice to Enhance Mental Health for Autistic People: A Scoping Review.” American Journal of Occupational Therapy 77, no. 2: 7702185060. 10.5014/ajot.2023.050074.

[jocn70494-bib-0069] Ní Riain, M. , and S. Wickham . 2023. “She Just Makes It Easier… the Impact of Having a Dedicated Nursing Role in Supporting People With Intellectual Disability When Accessing Acute Hospitals, From the Perspective of Their Support Staff, the Irish Context.” Journal of Intellectual Disabilities 28: 17446295231190252. 10.1177/17446295231190252. 37499117

[jocn70494-bib-0070] NICE . 2020. “Surveillance of Autism. Autism and Environmental Adaptions Within Inpatient Mental Health Units. NICE Guidelines CG128, CG141, CG170.”

[jocn70494-bib-0071] Noorlandt, H. W. , M. A. Echteld , I. Tuffrey‐wijne , et al. 2020. Shared Decision‐Making With People With Intellectual Disabilities in the Last Phase of Life: A Scoping Review. Wiley. 10.1111/jir.12774 32914520

[jocn70494-bib-0072] Nuffield Trust . 2016. “Reshaping the Workforce To Deliver The Care Patients Need.” https://www.nuffieldtrust.org.uk/research/reshaping‐the‐workforce‐to‐deliver‐the‐carepatientsneed.

[jocn70494-bib-0073] O'Hagan, B. , S. B. Krauss , A. J. Friedman , et al. 2023. “Identifying Components of Autism Friendly Health Care: An Exploratory Study Using a Modified Delphi Method.” Journal of Developmental and Behavioral Pediatrics 44, no. 1: e12–e18. 10.1097/DBP.0000000000001139. 36367772

[jocn70494-bib-0074] O'Leary, L. , S. A. Cooper , and L. Hughes‐McCormack . 2018. “Early Death and Causes of Death of People With Intellectual Disabilities: A Systematic Review.” Journal of Applied Research in Intellectual Disabilities: JARID 31, no. 3: 325–342. 10.1111/jar.12417 28984406

[jocn70494-bib-0075] Oulton, K. , J. Wray , A. Hassiotis , et al. 2019. Learning Disability Nurse Provision in Children's Hospitals: Hospital Staff Perceptions of Whether It Makes a Difference. Springer Science and Business Media LLC. 10.1186/s12887-019-1547-y PMC6558867 31182066

[jocn70494-bib-0096] Peters, M. D. J. , C. M. Godfrey , H. Khalil , P. McInerney , D. Parker , and C. B. Soares . 2015. “Guidance for Conducting Systematic Scoping Reviews.” International Journal of Evidence‐Based Healthcare 13, no. 3: 141–146. 10.1097/XEB.000000000000050. 26134548

[jocn70494-bib-0076] Peterson, J. , P. F. Pearce , L. A. Ferguson , and C. A. Langford . 2016. “Understanding Scoping Reviews: Definition, Purpose and Process.” Journal of the American Association of Nurse Practitioners 29, no. 1: 12–16. 10.1002/2327-6924.12380 27245885

[jocn70494-bib-0077] Pettersson, E. , B. M. Christensen , I. G. Berglund , and K. Huus . 2023. “Healthcare Professionals' Experiences of Situations During a Procedure With a Child With Autism Spectrum Disorder in the High‐Technology Environment.” Child: Care, Health and Development 49, no. 6: 1087–1095. 10.1111/cch.13119. 37009780

[jocn70494-bib-0078] Pridding, A. , and N. G. Procter . 2008. “A Systematic Review of Personality Disorder Amongst People With Intellectual Disability With Implications for the Mental Health Nurse Practitioner.” Journal of Clinical Nursing 17, no. 21: 2811–2819. 10.1111/j.1365-2702.2007.02269.x. 18624783

[jocn70494-bib-0079] Rowe, G. , and H. Nevin . 2013. “Bringing ‘Patient Voice’ Into Psychological Formulations of In‐Patients With Intellectual Disabilities, Autism Spectrum Disorder and Severe Challenging Behaviours: Report of a Service Improvement Pilot.” British Journal of Learning Disabilities 42, no. 3: 177–184. 10.1111/bld.12026.

[jocn70494-bib-0080] Samadi, S. A. 2008. “Comparative Policy Brief: Status of Intellectual Disabilities in the Islamic Republic of Iran.” Journal of Policy and Practice in Intellectual Disabilities 5, no. 2: 129–132.

[jocn70494-bib-0081] Samadi, S. A. , and R. McConkey . 2011. “Autism in Developing Countries: Lessons From Iran.” Autism Research and Treatment 2011: 145359. 10.1155/2011/145359. PMC3420542 22937242

[jocn70494-bib-0082] Simpson, S. 2020. Creating Accessible Healthcare Environments for People With Autism. Vol. 116. Emap Limited.

[jocn70494-bib-0083] Skills for Health and Health Education England . 2020. “Advanced Clinical Practice: Capabilities framework When Working with People Who Have a Learning Disability and/or Autism.”

[jocn70494-bib-0084] Squires, J. , R. E. Nickel , and D. Eisert . 1996. “Early Detection of Developmental Problems: Strategies for Monitoring Young Children in the Practice Setting.” Journal of Developmental and Behavioral Pediatrics 17, no. 6: 420–427. 10.1097/00004703-199612000-00008. 8960572

[jocn70494-bib-0085] Taua, C. , and T. Farrow . 2009. “Negotiating Complexities: An Ethnographic Study of Intellectual Disability and Mental Health Nursing in New Zealand.” International Journal of Mental Health Nursing 18, no. 4: 274–284. 10.1111/j.1447-0349.2009.00610.x. 19594647

[jocn70494-bib-0086] Tricco, A. C. , E. Lillie , W. Zarin , et al. 2018. “PRISMA Extension for Scoping Reviews (PRISMA‐ScR): Checklist and Explanation.” Annals of Internal Medicine 169, no. 7: 467–473. 10.7326/M18-0850. 30178033

[jocn70494-bib-0087] Unigwe, S. , C. Buckley , L. Crane , L. Kenny , A. Remington , and E. Pellicano . 2017. “GPs Confidence in Caring for Their Patients on the Autism Spectrum: An Online Self‐Report Study.” British Journal of General Practice 67, no. 659: e445–e452. 10.3399/bjgp17X690449 PMC5442960 28483821

[jocn70494-bib-0088] Urbanowicz, A. , T. Parkin , K. Van Dooren , S. Girdler , M. Ciccarelli , and N. Lennox . 2020. The Experiences, Views, and Needs of Health Professionals Who Provide Care to Adults on the Autism Spectrum. Informa UK Limited.

[jocn70494-bib-0089] Whittemore, R. , and K. Knafl . 2005. “The Integrative Review: Updated Methodology.” Journal of Advanced Nursing 52, no. 5: 546–553. 10.1111/j.1365-2648.2005.03621.x. 16268861

[jocn70494-bib-0092] Woo, B. F. Y. , J. X. Y. Lee , and W. W. S. Tam . 2017. “The Impact of the Advanced Practice Role on Quality of Care, Clinical Outcomes, Patient Satisfaction, and Cost in the Emergency and Critical Care Settings: A Systematic Review.” Human Resources for Health 15, no. 1: 1–122. 10.1186/s12960-017-0237-9 PMC5594520 28893270

[jocn70494-bib-0090] World Health Organisation . 2023. Technical Brief on Strengthening the Nursing and Midwifery Workforce to Improve Health Outcomes. WHO.

